# Effect of Self-Measuring Blood Pressure Program on Hypertension Control: Analysis by Diabetes Status, Age, Gender, and Race in Rural Arizona

**DOI:** 10.3390/clinpract14060208

**Published:** 2024-12-05

**Authors:** Joy Luzingu, Aminata Kilungo, Randall Flores, Zoe Baccam, Tenneh Turner-Warren, Thelma Reis, Babasola Okusanya, John Ehiri

**Affiliations:** 1 Department of Epidemiology and Biostatistics, Mel and Enid Zuckerman College of Public Health, The University of Arizona, 1295 N Martin Ave, Tucson, AZ 85724, USA; joyluzingu@arizona.edu; 2 Department of Community, Environment and Policy, Mel and Enid Zuckerman College of Public Health, The University of Arizona, 1295 N Martin Ave, Tucson, AZ 85724, USA; paminata@arizona.edu; 3 Chiricahua Community Health Centers, Inc., 1111 F Ave, Douglas, AZ 85607, USA; rflores@cchci.org; 4 School of Social Work, Arizona State University, 411 N Central Ave #800, University Center Building, Phoenix, AZ 85004, USA; zoebaccam@arizona.edu; 5 Bureau of Chronic Diseases and Health Promotion, Arizona Department of Health Services, Phoenix, AZ 85007, USA; tenneh.turner-warren@azdhs.gov (T.T.-W.); thelma.okotie@azdhs.gov (T.R.); 6 Health Promotion Sciences Department, Mel and Enid Zuckerman College of Public Health, The University of Arizona, 1295 N Martin Ave, Tucson, AZ 85724, USA; bokusanya@arizona.edu

**Keywords:** self-measuring blood pressure, hypertension, diabetes, rural Arizona

## Abstract

Background: Rural areas face numerous health challenges, including workforce shortages, limited training opportunities, and delayed care. These disparities can be mitigated by self-management interventions for diseases such as hypertension. This study assessed the implementation of a Self-Measuring Blood Pressure (SMBP) program in rural Arizona, documenting its barriers and patient experiences. Methods: In this before-after study, participants were loaned a digital device which they used to self-measure and record blood pressure (BP) over 1 week or more for hypertension diagnosis or 4 weeks or more for monitoring. Blood pressure (BP) control was assessed per the guidelines of the American Heart Association and American Diabetes Association. BP changes between baseline and post-program were assessed using paired-Student t tests. Effect modification by diabetes was analyzed using stratification. Results: Among 740 participants, significant associations were found with gender, age, and controlled BP among non-diabetic patients. Post-intervention, 63.4% of diabetic patients showed controlled BP, and 25.7% of non-diabetic patients had controlled BP, with higher control rates among females and older age groups (60–79 years). Baseline mean SBP was 148.3 ± 19.6 mmHg, improving to 133.9 ± 14.6 mmHg; baseline DBP was 88.5 ± 33.6 mmHg, improving to 83.4 ± 9.6 mmHg. Conclusions: The SMBP program effectively controlled BP, highlighting the value of combining clinical care with telemonitoring.

## 1. Introduction

Rural areas face many health challenges, such as health workforce shortage, limited rural health training opportunities, and delayed care [1,2,3,4]. This exacerbates health disparities, especially among low-income individuals and older adults [5]. Rural-dwelling older adults have a higher prevalence of chronic diseases compared to their urban counterparts [6]. For instance, a study that assessed medication therapy management between individuals in rural Arizona counties found that urban dwellers had significantly lower average systolic blood pressure (SBP) values at follow-up than those from rural communities [7]. Adequate self-management is important for effective treatment of hypertension. In the context of scarcity of health resources in rural communities, mobile technology, including self-measurement of blood pressure (SMBP), can help to bridge gaps in health service provision and follow-ups. Self-measurement of blood pressure (SMBP), also called “Home blood pressure monitoring”, is the measurement of blood pressure (BP) by a person outside of a clinical setting at their home. Monitoring BP through self-measurement plays an important role in managing hypertension and is a valuable complement to assessing and treating hypertension in the United States [8]. Self-measurement of blood pressure (SMBP) offers many benefits similar to those obtained with ambulatory BP monitoring, including a greater number of readings, absence of the white-coat syndrome effects, and elimination of the observer bias when an automated device is used. Additionally, SMBP can enhance adherence to antihypertensive medications and decrease the frequency of healthcare visits necessary for diagnosis and treating hypertension. This method also presents a more cost-effective option for BP monitoring [9]. Several studies have been published on SMBP. For example, a systematic review of 24 studies focusing on SMBP in the context of mobile health (mHealth) found that mHealth SMBP interventions were effective in BP control [10]. There is limited knowledge regarding the current prevalence of SMBP device ownership and frequency of use among patients and healthcare providers in the US, especially among minority and underserved communities [8]. Furthermore, its effectiveness in such areas has yet to be demonstrated. To improve BP control from 64.2% (according to the UDS data of 2019) to ≥80% by the end of 2023, the Chiricahua Community Health Centers (CCHCI) in Arizona implemented SMBP to diagnose hypertension among adults aged 18–85 years old, and to determine if there is BP control for those with hypertension. The intended benefits of the program were to improve the accuracy of diagnosing hypertension, better manage patient BP, and help patients adhere to non-pharmaceutical and pharmaceutical treatment and recommendations. However, there may be barriers that could impede the success of this program. For example, some patients may not be willing to participate in the program, or there may be other limitations such as low levels of literacy, including health and technology literacy, especially for the elderly population. The objectives of the research were to assess the implementation of SMBP interventions and document its impact, barriers, and patients’ experience.

## 2. Materials and Methods

### 2.1. Study Population

The study population included adults aged 18 or older who received health care services at the CCHCI between September 2021 and March 2023 and either had a current diagnosis of hypertension or were suspected to have hypertension due to elevated BP readings.

#### 2.1.1. Quantitative Study Design

A before–after study was conducted at the CCHCI in Chiricahua, Arizona between September 2021 and March 2023.

a. Inclusion criteria:

Participants were included in the study if they were 18–85 years old and either had an existing diagnosis of hypertension (inclusion criterion 1) or were suspected to have hypertension (or undiagnosed hypertension) (inclusion criterion 2).

b. Exclusion criteria:

The study did not include anyone who did not meet the inclusion criteria.

If the patient was enrolled in the program for inclusion criterion 1, they were followed up for four weeks. However, the length of follow-up could be longer, depending on the provider’s judgment. If the patient was enrolled per inclusion criterion 2, they were seen in one week to confirm or rule out hypertension. If the diagnosis of hypertension was ruled out, these subjects were not included in the study. Participants with confirmed hypertension were followed up for at least four weeks.

Patients aged 18–85 years diagnosed with hypertension, irrespective of having diabetes, were recruited. Those who accepted to enroll in the program were loaned a cellular device to self-measure their BP. The BP measurement device was a Smart Meter brand, included on the list of US BP-validated BP cuffs [11]. Participants were given the device for one or more weeks to monitor their BP. If hypertension was not controlled, participants could borrow the device for an extended period of four weeks or more. Per protocol, at the start of the SMBP intervention, participants were educated on how to use the device to check their BP accurately. They were advised on what their BP goal was. Also, they checked their BP by taking two readings at least one minute apart twice daily (two readings in the morning and two in the evening) per American Heart Association (AHA) recommendations [12]. The objective of the SMBP program was to improve BP control from 64.2% (2019 UDS data) to ≥80% by the end of 2023. All four daily BP recordings were automatically remotely recorded on a “TimeDoc” portal because of the link between the BP cuff and TimeDoc^®^. TimeDoc^®^ is a web-based portal that connects patients and their providers by combining remote care services, an electronic health record (EHR)-integrated dashboard, and easy-to-use devices such as SMBP devices or cellular-enabled blood glucose meters [13]. At the end of the loan period, each participant returned the device, with the information on the device and return date saved in an inventory log.

#### 2.1.2. Qualitative Study Component

The qualitative study involved adults who refused to participate in the SMBP program, with the aim of better understanding the barriers, limitations, and reasons for refusal. Fifty people were surveyed via REDCap. The survey captured demographic (age, race, ethnicity) information and reasons for not participating in SMBP.

### 2.2. Measures

The baseline systolic and diastolic BP was measured using an aneroid (manual) or oscillometric (digital) BP device, depending on what was routine at the clinic sites. The end-point BP was the average of the SBP and DBP that were self-measured and recorded on the device.

The condition for controlled BP was BP < 130/80 mmHg for non-diabetic participants, as recommended by the AHA guidelines [14], and BP < 140/90 mmHg for diabetic participants, as recommended by the American Diabetes Association guidelines [15].

At baseline, a preexisting diagnosis of diabetes was confirmed from her, or screening for diabetes was confirmed with a hemoglobin A1C ≥ 6.5% or fasting plasma glucose ≥126 mg/dL.

The percentage of participants achieving BP control was calculated by dividing the number of non-diabetic and diabetic participants who met the respective control criteria by the total number of non-diabetic and diabetic participants, respectively, and multiplying these fractions by 100.

### 2.3. Data Analysis

For the quantitative part of the study, variables were summarized using simple frequencies and percentages for categorical variables and medians with ranges for the age continuous variable. The associations between controlled BP and socio-demographic characteristics were assessed using the Chi-square test. A paired t-test was used to compare baseline and post-program SBP/DBP (measured with TimeDoc^®^) for all the data to determine the impact of SMBP on BP control. To assess effect modification by diabetes in the relation between SMBP and BP control, the same comparison within the subgroups of participants with and without diabetes was made. Statistical analyses were performed using Stata version 18.0., STATA Corp LL, College Station, TX, USA, and a *p*-value < 0.05 was considered statistically significant.

For the qualitative part of the study, demographic variables were summarized descriptively. The qualitative data were reported directly from the surveys themselves. Analyses were performed using Stata version 18.0 and Microsoft Excel, version 16.91.

### 2.4. Ethical Approval

This study received approval from The University of Arizona Institutional Review Board (IRB) under protocol number 2001288660 on 07 February 2020 and followed the ethical standards of the Declaration of Helsinki. 

## 3. Results

**Figure 1:**
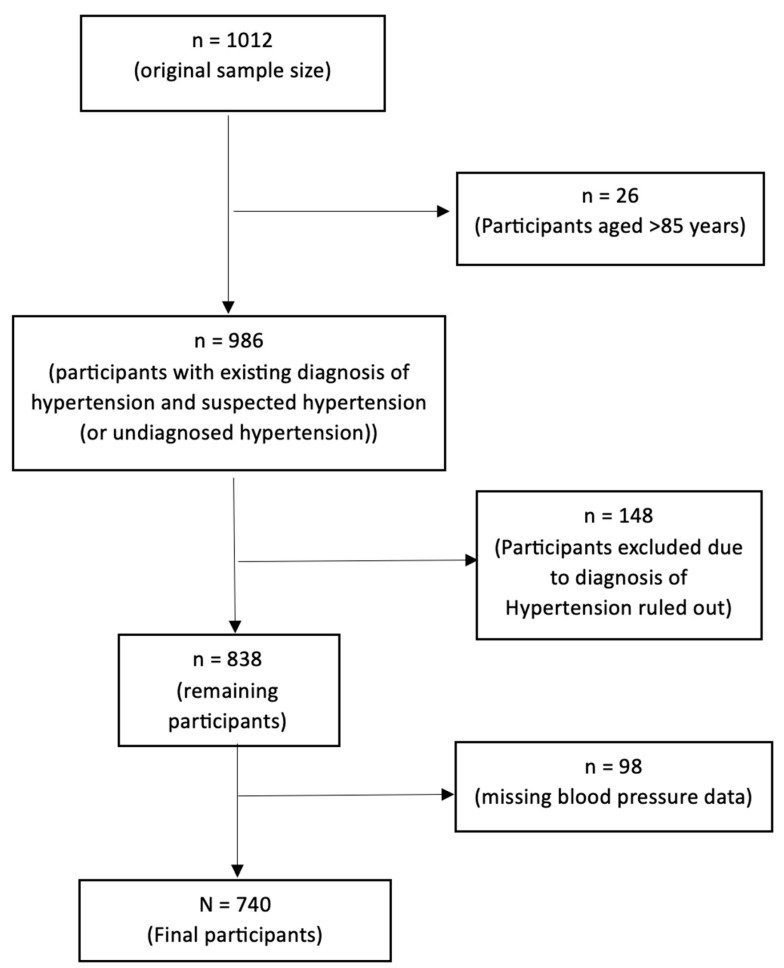
Participant flow diagram.

The SMBP program had 1012 participants enrolled. Twenty-six (26) participants were excluded because they were older than 85 years. An additional 148 participants were excluded because the diagnosis of hypertension was ruled out, and 98 participants were excluded because baseline SBP and DPB and/or TimeDoc^®^ SBP and DBP were unavailable. Therefore, only 740 participants were included in the quantitative study. See Figure 1 for the participants’ inclusion and Table 1 for the sociodemographic characteristics. All fifty participants who refused to participate in the SMBP study were included for the qualitative study to elucidate the reasons for non-participation.

The median age of the participants was 62.0, with a range of 22–85 years, with more than half of the participants being females. Data on ethnicity were missing for about a third of the participants. The majority (73.4%) of participants were white (Table 1).

At the entry of the SMBP program, 86.9% of participants were diagnosed with hypertension, while only 10.5% had concurrent hypertension diagnoses according to the SMBP. A third (29.6%) of participants had diabetes. At the end of the SMBP intervention, when the device was returned, BP values were controlled in 25.7% of non-diabetic and 63.4% of diabetic patients when compared to the baseline (Table 2).

Table 3 shows that there were statistically significant associations between control of BP and gender and age groups among non-diabetic patients. Among those non-diabetic patients with controlled BP, 64.2% were female. Increasing age was associated with a higher prevalence of controlled BP as of age 50 up to an age of 69 years. The proportion of non-diabetic patients whose BP was controlled was higher in the 60–69- and 70–79-years age groups and lower in the oldest (80–85-years) and 40–49-years age groups.

Comparisons of mean SBP and DBP between the baseline and end of the program are presented in Table 4. There was a decrease in mean SBP in both diabetic and non-diabetic patients. For all participants, the mean SBP values were 148.3 ± 19.6 mmHg at baseline and 133.9 ± 14.6 mmHg at the end of the program; the DBP values at baseline and post-program were 88.5 ± 33.6 mmHg and 83.4 ± 9.6 mmHg, respectively.

Among non-diabetic patients, the mean SBP at baseline was 148.2 ± 19.0 mmHg, while after the program, it was 133.9 ± 14.1 mmHg; the mean DBP was 88.4 ± 12.0 mmHg at baseline and 84.4 ± 9.7 mmHg at the end of the program.

Among diabetic patients, the mean SBP was 148.4 ± 20.8 mmHg and 133.7 ± 15.8 mmHg at baseline and post-program, respectively. Also, the DBP was 88.9 ± 59.0 mmHg and 80.8 ± 8.6 mmHg at baseline and post-program, respectively. These decreases in BP were statistically significant in all associations.

Table 5 demonstrates that changes in blood pressure between post-program and baseline were statistically significant after stratifying for age groups and race (white versus non-white) in different subgroups, except for DBP in the 18–39 and 80–85 age groups.

Table 6 demonstrates that changes in blood pressure between post-program and baseline were statistically significant after stratifying for race (white versus non-white) within the subgroups of participants with and without diabetes, except for DBP among non-white participants with diabetes.

Table 7 shows that changes in blood pressure between post-program and baseline were statistically significant after stratifying for gender within the subgroups of participants with and without diabetes, except for DBP among male participants with diabetes.

Table 8 displays that changes in blood pressure between post-program and baseline were statistically significant after stratifying for age groups within the subgroups of participants with and without diabetes, except for DBP participants aged 18–39 and 80–85 with diabetes and among participants aged 80–85 years old without diabetes.

The median age of those who participated in the qualitative research was 70 years (range = 21–89). Thirty-nine (39) participants were white (78%). Hispanics represented 48% of the sample (Table 9).

Table 10 shows that the main reason for not participating in the program was that persons were not interested (76%), followed by other reasons (22%). One person (2%) declined to participate because they were already in a similar program.

Among the 38 persons who were not interested in the program, 10 provided comments on their lack of interest in participating, namely:

- Not wanting to check BP regularly and considering that their BP was already controlled.
- Rejection should be considered as an initial rejection and participant might consider participating later.
- Patient was not sure if they wanted to stay in the facility.
- Worried about knowing that their BP was high.
- Patient had a stable BP and did not think they needed the program.
- Patient would think about the program.
- Patient was already seeing another primary care provider.
- Patient stated that they had their BP device and were not interested even though they were explained that the program’s device was different.
- Patient wanted to purchase their own BP device.
- Time constraint with work.

## 4. Discussion

The objectives of this research were to assess the impact, barriers, and patients’ experience of the SMBP intervention. The main finding of this study was that the SMBP program in Chiricahua led to a significant decrease in the BP of both diabetic and non-diabetic participants. Among non-diabetic patients, the goal of a BP < 130/ 80 mmHg was not attained, as the mean post-program SBP and DBP were slightly higher than the AHA recommendations. Our findings align with a meta-analysis of randomized controlled trials which showed that regular implementation of home BP telemonitoring (HBPT), defined as the remote transmission of BP values measured at home and transmitted to the clinician’s office or hospital using telehealth strategies [16], during a 6-month follow-up period was associated with higher reductions in BP [17]. It is important to mention that HBPT should not be overvalued, since the strength of evidence for the benefit of telemedicine in hypertension management compared with usual care shows a moderate level of reduction in BP [18]. We also found that, among non-diabetic patients, the prevalence of controlled BP was associated with gender (higher rate of hypertension control in women than in men) and was higher in the 60–69- and 70–79-years age groups. Similar results were found concerning a lower rate of control of hypertension in men than in women [19,20]. Young adult women are more likely to have their BP checked than young adult men (e.g., for pregnancy or gynecology care) [21]. Therefore, health providers should pay particular attention to the treatment and management of hypertension among young adult men. Concerning the prevalence of controlled BP differences in age groups, our findings are similar to those from another study which found that controlled BP was more likely in the 45–64 age group (49.7%) compared to the 18–44 age group (36.7%) [22]. Older young adults may have accumulated more knowledge and experience in managing their health, including understanding the importance of checking their BP for overall well-being. We also found that SMBP essentially led to significant decreases in BP across the whole sample after stratifying by age groups and race (white versus non-white). Additionally, within subgroups of participants with and without diabetes, significant decreases were observed after stratifying by race (white versus non-white), gender, and age groups. These findings demonstrate that the program was effective in decreasing BP even after accounting for stratification by demographic and diabetes status.

The findings of the qualitative component of the study, where a majority (76%) refused to participate in the SMBP because they were not interested, was supported by the results from a larger study, in which 88% (10,010/11,399) of participants declined to participate in BP self-management research. This large clinical trial conducted in the UK by McManus et al. assessed a digital tool for hypertension management by integrating BP self-monitoring and self-management support. It found that, among those who declined to participate in the study, 2426 (24%) gave their reasons for refusal, including lack of internet access (982, 41%) and not wanting to be part of a research trial (617, 25%), amongst other reasons [23]. The reasons for refusal differed in both studies, probably due to the differences in sample sizes, demographics, and settings. To improve adherence to SMBP programs, strategies such as emphasizing the long-term benefits of regular blood pressure monitoring, highlighting the unique features of the loaned BP cuffs, and conducting follow-up outreach could help convert initial refusals into future participants. Additionally, providing educational support may reinforce the importance and value of self-monitoring.

### Strengths and Limitations

This study had some strengths. First, the study was conducted among relatively older adults, with more than 50% (56.4%) of participants aged 60 years and above. Nearly 60% of the population has hypertension by the age of 60 years [24], so our study yielded results from persons more vulnerable to hypertension. Second, the study not only confirmed findings from previous studies, but also brought insight into self-management of hypertension in rural Arizona, where access to care might be limited. However, the study had some limitations too. First, the lack of information on other reasons that people did not want to participate in the SMBP program does not allow us to have a better understanding of factors that impede people in adopting self-measurement of their BP. Second, the study only focused on a rural area in Arizona, which may limit the generalizability of its findings. Third, although we noticed control of BP among the participants, we cannot certainly attribute these changes to the SMBP program only, as we did not have information about participants’ antihypertensive medication regimens, changes in prescriptions, or medication adherence during the study period. This is particularly important as SMBP programs may improve medication adherence, which could be a key mechanism for BP reduction [25]. Also, the absence of biochemical parameters (such as pre- and post-prandial glucose levels and lipid profiles), history of concomitant diseases, lifestyle factors (such as smoking and alcohol consumption), dietary habits, medication usage, and comprehensive lifestyle variables is a notable limitation of this study. Including these variables could have enriched our understanding of the influence of metabolic status and lifestyle on the effectiveness of the SMBP program, particularly among participants with diabetes. Fourth, the study’s before–after design makes it challenging to draw causal inferences about the SMBP program since there was no comparison group. Fifth, in this study, baseline BP measurements were collected using different devices across clinic sites, either aneroid (manual) or oscillometric (digital), depending on equipment availability at each location. Post-program measurements, however, were uniformly collected with a standardized digital device provided to all participants. We recognize this variation in measurement methods as a significant limitation of our study. The device inconsistency may have introduced variability in the results, potentially affecting the accuracy and comparability of the BP changes reported. Readers should consider this methodological constraint when interpreting the effectiveness of the SMBP program as reported in our findings. Finally, the study did not report findings from the qualitative design in an appropriate way. This was because qualitative data were not collected from focus groups or in-depth interviews, but rather from an online survey which did not allow for in-depth discussion to understand why some participants were not interested.

Future research should target larger populations and use appropriate qualitative data collection methods to conduct a better assessment of SMBP across the country in both a quantitative and qualitative fashion. To enhance the validity and reliability of future research on SMBP monitoring, we recommend using the same type of BP measurement device throughout the study. Future studies should make sure that all baseline and follow-up BP readings are collected with the same model and type of device to reduce variability and improve the comparability of the results. Also, it would be beneficial to include a broader range of biochemical parameters, such as pre- and post-prandial glucose levels; lipid profiles; and detailed information on concomitant disease histories, medications, and lifestyle factors such as smoking and alcohol consumption in future studies. Such data would allow to examine the nuanced effects of SMBP across various metabolic and lifestyle backgrounds, especially in diabetic and non-diabetic patients. Finally, the assessment of genetic markers associated with hypertension and cardiovascular diseases could offer profound insights into the personalized effectiveness of SMBP programs.

## 5. Conclusions

This study demonstrated that the SMBP program in Chiricahua, Arizona was effective in controlling BP in enrolled participants. Also, it provided insight into the reasons why some individuals did not participate in the program. These results demonstrate the importance of such a program in rural areas, where access to healthcare may be limited for many reasons. The findings of this study call for the integration of clinical care with telemonitoring of BP to achieve better health outcomes for rural populations with limited access to services.

## Figures and Tables

**Table 1:** Socio-demographic characteristics of participants (N = 740).

Socio-Demographic Characteristics	N (%)
Age in years, Median (min, max)	62.0 (22, 85)
Age groups (in years)	
18–39	86 (11.6)
40–49	75 (10.1)
50–59	162 (21.9)
60–69	214 (28.9)
70–79	164 (22.2)
80+	39 (5.3)
Gender	
Female	405 (54.7)
Male	335 (45.3)
Race	
White	543 (73.4)
Pacific Islander or native Hawaiian	6 (0.8)
Asian	10 (1.4)
Native American	10 (1.4)
Black	33 (4.4)
Hispanic	17 (2.3)
Prefer not to answer	5 (0.7)
Declined to specify	85 (11.5)
Prefer not to disclose race	3 (0.4)
Multiracial	1 (0.1)
Missing	27 (3.6)
Ethnicity	
Hispanic or Latino	287 (38.8)
Not Hispanic or Latino	158 (21.4)
Declined to specify	51 (6.9)
Unknown/Not reported	5 (0.7)
Missing	239 (32.3)

**Table 2:** Clinical characteristics of participants (N = 740).

Characteristics	N (%)
Existing diagnosed hypertension	
No	97 (13.1)
Yes	643 (86.9)
Hypertension diagnosed with SMBP	
No	662 (89.5)
Yes	78 (10.5)
Diagnosed diabetes	
No	521 (70.4)
Yes	219 (29.6)
Controlled BP among non-diabetics	
No	387 (74.3)
Yes	134 (25.7)
Controlled BP among diabetics	
No	79 (36.1)
Yes	139 (63.4)
Missing	1 (0.5)

**Table 3:** Association between control of BP and sociodemographic characteristics of participants.

Variables	Controlled BP Among Non-Diabetic Patients, n (%)			Controlled BP Among Diabetic Patients, n (%)		
	No	Yes	*p* Value	No	Yes	*p* Value
Gender			0.011			0.548
Female	199 (51.4)	86 (64.2)		41 (51.9)	78 (56.1)	
Male	188 (48.6)	48 (35.8)		38 (48.1)	61 (43.9)	
Age categories in years			0.024			0.550
18–39	51 (13.2)	23 (17.2)		3 (3.8)	9 (6.5)	
40–49	50 (12.9)	13 (9.7)		5 (6.3)	7 (5.0)	
50–59	92 (23.7)	21 (15.7)		17 (21.5)	32 (23.0)	
60–69	99 (25.6)	44 (32.8)		22 (27.9)	49 (35.3)	
70–79	66 (17.1)	30 (22.4)		30 (38.0)	37 (26.6)	
80–85	29 (7.5)	3 (2.2)		2 (2.5)	5 (3.6)	
Race			0.253			0.269
White	277 (74.6)	105 (81.4)		52 (69.3)	109 (79.5)	
Pacific Islander or native Hawaiian	6 (1.6)	0 (0.0)		0 (0.0)	0 (0.0)	
Asian	4 (1.1)	3 (2.3)		0 (0.0)	2 (1.5)	
Native American	5 (1.4)	2 (1.6)		1 (1.3)	2 (1.5)	
Black	17 (4.6)	4 (3.1)		5 (6.7)	7 (5.1)	
Hispanic	9 (2.4)	1 (0.8)		3 (4.0)	4 (2.9)	
Prefer not to answer	2 (0.5)	1 (0.8)		2 (2.7)	0 (0.0)	
Declined to specify	50 (13.5)	11 (8.5)		11 (14.7)	13 (9.5)	
Prefer not to disclose race	1 (0.3)	2 (1.5)		0 (0.0)	0 (0.0)	
Multiracial	0 (0.0)	0 (0.0)		1 (1.3)	0 (0.0)	
Ethnicity			0.601			0.432
Hispanic or Latino	139 (54.7)	56 (58.9)		28 (54.9)	64 (64.0)	
Not Hispanic or Latino	80 (31.5)	31 (32.6)		19 (37.3)	27 (27.0)	
Declined to specify	31 (12.2)	7 (7.4)		4 (7.8)	9 (9.0)	
Unknown/Not reported	4 (1.6)	1 (1.1)		0 (0.0)	0 (0.0)	

**Table 4:** Comparisons of baseline and TimeDoc^®^ post-program SBP and DBP.

Variable	Baseline	TimeDoc^®^ Post-SMBP	Mean Difference (95% CI) in mmHg	*p* Value
All participants				
Systolic BP (mean ± SD) in mmHg	148.3 ± 19.6	133.9 ± 14.6	−14.4 (−15.8; −13.0)	0.000
Diastolic BP (mean ± SD) in mmHg	88.5 ± 33.6	83.4 ± 9.6	−5.2 (−7.6; −2.8)	0.000
Among non-diabetic patients				
Systolic BP (mean ± SD) in mmHg	148.2 ± 19.0	133.9 ± 14.1	−14.3 (−15.9; −12.7)	0.000
Diastolic BP (mean ± SD) in mmHg	88.4 ± 12.0	84.4 ± 9.7	−3.9 (−4.9; −2.9)	0.000
Among diabetic patients				
Systolic BP (mean ± SD) in mmHg	148.4 ± 20.8	133.7 ± 15.8	−14.7 (−17.5; −11.8)	0.000
Diastolic BP (mean ± SD) in mmHg	88.9 ± 59.0	80.8 ± 8.6	−8.1 (−15.9; −0.3)	0.0212

**Table 5:** Comparisons of baseline and TimeDoc^®^ post-program SBP and DBP within age groups and race (white vs. non-white).

Variable	n	Baseline (Mean ± SD)	TimeDoc^®^ Post-SMBP (Mean ± SD)	Mean Difference (95% CI)	*p* Value
Age groups					
18–39					
SBP, mmHg	86	141.9 ± 14.2	130.2 ± 12.5	−11.7 (−15.1; −8.3)	0.000
DBP, mmHg	86	103.0 ± 91.8	86.2 ± 9.3	−16.9 (−36.6; 2.8)	0.05
40–49					
SBP, mmHg	75	145.1 ± 16.4	132 ± 12.0	−12.4 (−16.4; −8.3)	0.000
DBP, mmHg	75	91.8 ± 10.3	87.8 ± 9.0	−4.0 (−6.6; −1.5)	0.001
50–59					
SBP, mmHg	162	147.0 ± 20,6	132.3 ± 14.6	−14.7 (−17.8; −11.6)	0.000
DBP, mmHg	162	88.7 ± 11.7	86.0 ± 9.0	−2.7 (−4.5; −0.9)	0.002
60–69					
SBP, mmHg	214	148.9 ± 20.5	134.8 ± 16.2	−14.2 (−17.0; −11.4)	0.000
DBP, mmHg	214	86.8 ± 12.5	82.9 ± 9.9	−3.8 (−5.5; −2.2)	0.000
70–79					
SBP, mmHg	164	153.3 ± 19.8	135.2 ± 13,9	−18.1 (−21.2; −15.0)	0.000
DBP, mmHg	164	84.0 ± 11.2	80.0 ± 8.0	−4.3 (−6.1; −2.7)	0.000
80–85					
SBP, mmHg	39	149.5 ± 19.9	140.4 ± 14.2	−9.1 (−15.8; −2.4)	0.005
DBP, mmHg	39	77.9 ± 12.8	75.5 ± 7.1	−2.4 (−6.0; 1.3)	0.09
Race					
White					
SBP, mmHg	543	148.4 ± 19.5	133.7 ± 14.4	−14.7 (−16.3; −13.0)	0.000
DBP, mmHg	543	86.7 ± 11.8	82.8 ± 9.3	−3.9 (−4.8; −3.0)	0.000
Non-white					
SBP, mmHg	162	148.2 ± 20.5	134.3 ± 1.2	−13.9 (−17.2; −10.7)	0.000
DBP, mmHg	162	94.6 ± 68.0	84.9 ± 10.1	−9.7 ± (−20.2; 0.907)	0.036

Category “missing” for race not shown. To create the binary race variable, categories “Prefer not to disclose race” and “Prefer not to answer” were considered as missing values, the other categories except “White” were categorized as “Non = white”.

**Table 6:** Comparisons of baseline and TimeDoc^®^ post-program SBP and DBP within subgroups of participants with and without diabetes by race (white vs. non-white).

Measure	n	Baseline (Mean ± SD	TimeDoc^®^ Post-SMBP (mean ± SD)	Mean Difference (95% CI)	*p* Value
Diabetes					
White					
SBP, mmHg	161	148.3 ± 21.5	133.3 ± 15.9	−15.1 (−18.5; −11.7)	0.000
DBP, mmHg	161	84.4 ± 12.1	80.0 ± 8.4	−4.4 (−6.2; −2.6)	0.000
Non-white					
SBP, mmHg	50	148.6 ± 19.5	133.7 ± 15.1	−15.0 (−20.8; −9.2)	0.000
DBP, mmHg	50	102.9 ± 121.2	82.4 ± 9.0	−20.5 (−54.8; 13.8)	0.117
No Diabetes					
White					
SBP, mmHg	382	148.4 ± 18.6	134.0 ± 13.7	−14.5 (−16.3; −12.6)	0.000
DBP, mmHg	382	87.6 ± 11.5	83.9 ± 9.4	−3.7 (−4.7; −2.6)	0.000
Non-white					
SBP, mmHg	112	148.0 ± 21.0	134.6 ± 15.2	−13.4 (−17.4; −9.5)	0.000
DBP, mmHg	112	90.9 ± 13.5	86.0 ± 10.4	−4.8 (−7.6; −2.0)	0.001

Category “missing” for race not shown. To create the binary race variable, categories “Prefer not to disclose race” and “Prefer not to answer” were considered as missing values, the other categories except “White” were categorized as “Non = white”.

**Table 7:** Comparisons of baseline and TimeDoc^®^ post-program SBP and DBP within subgroups of participants with and without diabetes by gender.

Measure	n	Baseline (Mean ± SD	TimeDoc^®^ Post-SMBP (Mean ± SD)	Mean Difference (95% CI)	*p* Value
Diabetes					
Female					
SBP, mmHg	120	149.5 ± 20.8	132.9 ± 15.9	−16.7 (−20.7; −12.6)	0.000
DBP, mmHg	120	85.9 ± 11.3	79.9 ± 7.7	−6.0 (−8.0; −4.0)	0.000
Male					
SBP, mmHg	99	147.1 ± 20.9	134.8 ± 15.6	−12.3 (−16.2; −8.4)	0.000
DBP, mmHg	99	92.5 ± 87.0	82.0 ± 9.5	−10.5 (−27.8; 6.7)	0.113
No Diabetes					
Female					
SBP, mmHg	285	146.3 ± 19.0	132.0 ± 14.6	−14.3 (−16.3; −12.0)	0.000
DBP, mmHg	285	87.3 ± 11.6	83.0 ± 9.7	−4.3 (−5.6; −2.9)	0.000
Male					
SBP, mmHg	236	150.0 ± 18.8	136.3 ± 13.1	−14.3 (−16.6; −12.0)	0.000
DBP, mmHg	236	89.7 ± 12.4	86.2 ± 9.5	−3.5 (−5.1; −2.0)	0.000

**Table 8:** Comparisons of baseline and TimeDoc^®^ post-program SBP and DBP within subgroups of participants with and without diabetes by age groups.

Measure	n	Baseline (Mean ± SD	TimeDoc^®^ Post-SMBP (Mean ± SD)	Mean Difference (95% CI)	*p* Value
Diabetes					
18–39					
SBP, mmHg	12	144.6 ± 18.8	128.0 ± 16.2	−16.6 (−30.8; −2.4)	0.013
DBP, mmHg	12	163.0 ± 245.1	85.1 ± 10.4	−77.9 (−233.2; −77.3)	0.146
40–49					
SBP, mmHg	12	150.7 ± 18.9	135.6 ± 15.8	−15.1 (−27.2; −3.0)	0.009
DBP, mmHg	12	97.8 ± 10.7	86.1 ± 9.5	−11.8 (−19.7; −3.8)	0.003
50–59					
SBP, mmHg	49	146.2 ± 16.9	131.2 ± 15.8	−15.0 (−21.0; −9.1)	0.000
DBP, mmHg	49	87.2 ± 10.2	82.8 ± 8.0	−4.3 (−7.4; −1.2)	0.003
60–69					
SBP, mmHg	71	145.5 ± 22.6	134.1 ± 17.0	−11.4 (−16.7; −6.0)	0.000
DBP, mmHg	71	82.8 ± 11.1	80.4 ± 8.7	−2.5 (−5.2; 0.3)	0.040
70–79					
SBP, mmHg	68	153.4 ± 21.7	136.0 ± 14.7	−17.4 (−22.6; −12.3)	0.000
DBP, mmHg	68	83.1 ± 11.4	78.6 ± 7.9	−4.5 (−7.1; −1.8)	0.000
80–85					
SBP, mmHg	7	147.7 ± 22.7	132.6 ± 12.2	−15.1 (−27.6; −2.7)	0.012
DBP, mmHg	7	76.0 ± 9.4	75.9 ± 5.4	−0.1 (−9.0; 8.5)	0.484
No Diabetes					
18–39					
SBP, mmHg	74	141.4 ± 13.4	130.5 ± 11.9	−10.9 (−14.3; −7.5)	0.000
DBP, mmHg	74	93.3 ± 9.0	86.4 ± 9.2	−7.0 (−9.7; −4.3)	0.000
40–49					
SBP, mmHg	63	144.0 ± 15.8	132.2 ± 11.3	−11.8 (−16.2; −7.5)	0.000
DBP, mmHg	63	90.7 ± 9.8	88.1 ± 8.9	−2.6 (−5.1; 0.03)	0.026
50–59					
SBP, mmHg	113	147.3 ± 22.1	132.8 ± 14.1	−14.5 (−18.2; −10.9)	0.000
DBP, mmHg	113	89.4 ± 12.3	87.4 ± 9.1	−2.0 (−4.3; 0.3)	0.040
60–69					
SBP, mmHg	143	150.7 ± 19.3	135.1 ± 15.9	−15.6 (−18.8; −12.3)	0.000
DBP, mmHg	143	88.7 ± 12.7	84.2 ± 10.3	−4.5 (−6.6; −2.5)	0.000
70–79					
SBP, mmHg	96	153.2 ± 18.4	134.7 ± 13.4	−18.6 (−22.6; −14.6)	0.000
DBP, mmHg	96	84.6 ± 11.0	80.4 ± 7.9	−4.3 (−6.5; −2.0)	0.000
80–85					
SBP, mmHg	32	149.8 ± 14.2	142.1 ± 14.2	−7.8 (−15.7; 0.1)	0.026
DBP, mmHg	32	78.3 ± 13.0	75.4 ± 7.5	−2.9 (−7.1; 1.3)	0.086

**Table 9:** Characteristics of qualitative component participants.

Characteristics	n (%)
Age in years, median (min, max)	70 (21, 89)
Race, n (%)	
White	39 (78.0)
Asian	1 (2.0)
Black or African American	2 (4.0)
Declined to specify	1 (2.0)
Multiracial	1 (2.0)
Prefer not to answer	1 (2.0)
Unknown	5 (10.0)
Ethnicity, n (%)	
Hispanic or Latino	24 (48.0)
Not Hispanic or Latino	16 (32.0)
Declined to specify	1 (2.0)
Unknown	9 (18.0)

**Table 10:** Reasons for rejection to participate in the SMBP (N = 50).

Rejection Reason	n (%)
In a similar program, n (%)	1 (2.0)
Not interested, n (%)	38 (76.0)
Other, n (%)	
Did not need it	1 (2.0)
Patients were no longer seeing a primary care physician in Chiricahua	8 (16.0)
Ended due to faulty machine	1 (2.0)
Patient refused BP cuff	1 (2.0)

## Data Availability

Data used in this study were de-identified and are available upon request from the corresponding author and the Chiricahua Community Health Centers.
